# Obstetrical Society of Philadelphia

**Published:** 1890-02

**Authors:** J. M. Baldy

**Affiliations:** Secretary


### OBSTETRICAL SOCIETY OF PHILADELPHIA.

DECEMBER 5, 1889.

Reported by J. M. BALDY, M. D., Secretary.

### MENORRHALGIA AND MENORRSPASM.

BY G. BETTON MASSEY, M. D.

The list of pathological views that have been advanced in accounting for what is usually called dysmenorrhea is somewhat extended, even when the term dysmenorrhea is restricted to the uterine type of painful menstruation, excluding ovarian and inflammatory pains and true neuralgia. Those most prevalent at the present time, I believe, are, on the one hand, the mechanical theory of obstruction from stenosis or flexion, which may be called the Marion Sims theory, and the parametritis theory of Schultze. It is not sufficiently well known that this latter observer has completely upset the first or obstructive theory of painful menstruation by demonstrating that a sound may be passed, during the crisis of a supposed example of accumulation, without encountering fluid. Such a view is also weakened by the examples of stenosis and anteflexion that occur without painful menstruation. Yet Schultze's theory of parametric inflammation as a cause, seems to me unsatisfactory. That it has failed of practical acceptance by those even who advocate it, is shown by their adherence to dilatation as a means of cure.

In that excellent picture of painful menstruation contributed by W. Gill Wylie to the "American System of Genecology," another pathological condition is suggested,—hyperesthesia of the endometrium. That an hyperesthetic condition of the cavity does exist in these cases, I think any one who has passed a sound into them will admit. The exclamations of pain when the internal os is passed are most characteristic, and, in cases where a proper gentleness has been observed, must be other than normal; but I do not think that the word hyperesthesia is sufficiently comprehensive as a designation of this condition. Dysmenorrhea, or difficult menstruation, is also but a partial description of the occurrence. In view of these facts I wish to present in brief to this society a new conception of the condition involved in painful menstruation as it has been suggested to me by recent clinical studies; and I also desire to propose a more useful name as a designation of the condition.

Abnormal pain at the menstrual period usually precedes the appearance of the flow, or it may follow a slight show, and be succeeded by a normal flow. As a rule, there is no flow at the moment that the pain is greatest. These facts have been the clinching arguments in the obstruction theory; but do they prove it? The absence of dilatation of the cavity above the point of apparent obstruction is significant. This, coupled with Schultze's observations, is fatal to the theory. The dependence of pain upon spasm, however, is clear, and the absence of flow, or slight flow, during the continuance of the pain only shows that the spasmodic condition of the uterus interferes with the excretory duties of the mucous membrane. Gastralgia, during the continuance of nervous dyspepsia, and simple intestinal colic, are analogous conditions. If I am right in this matter, the use of the word dysmenorrhea should be discontinued, as it forever suggests a mere mechanical condition. In its stead I propose the term *menorrhalgia* as a symptomatic designation that is etymologically in accord with associated terms, and does not tie us to a theory. If, again, it is believed that a given case of menorrhalgia is due to an inhibitory spasm, it should be called a *menorrspasm.*

This menorrspasm is usually accompanied by a permanently hyperesthetic condition of the endometrium, and is often indicated between periods by a spasmodic stricture of the internal os when an attempt at sounding is made. Exactly how much of this intermenstrual stenosis is spasmodic and how much fibrous, remains to be proven. The existence of the fibrous variety is, of course, undoubted; but the ease with which ether relaxes many canals sufficient to permit a dilator to be inserted indicates that they cannot be common, for, of course, an anesthetic could have no effect upon fibrous tissue.

Pathological anteflexion is also frequently found associated with menorrhalgia; but since the equal degree of this form of deviation may be found without pain, there can be no *essential* relation of cause and effect. The same may be said of endometritis and metritis. The frequency of menorrhalgia and its probable cause—menorrspasm—during a chronic metritis, without any evidence of stenosis, is an additional proof of the non-mechanical nature of this condition, as the inflammation would doubtless interfere with contraction, and aggravate spasm at the same time.

In presenting these conceptions as a novelty, I do not wish to be understood as claiming the idea of spasm as connected with painful menstruation. Such a condition has been conceded all along, and is well understood by the patients themselves when they speak of "cramps." But the contractions have been supposed to cause pain, because the flow was pent up. The spasm, as an inhibition of a normal excretion, has not been dwelt upon.

Menorrspasm, in brief, may be said to be a neuro-myotic storm of the uterine neuro-muscular apparatus, which renders the excretion of the menstrual fluid temporarily impossible. Its remote causes may be traced to all those influences in modern life which hinder the proper development of animal life in young women.

The treatment of the disease is both general and local. Many cases get well after regulation of the bodily functions and the correction of imperfect hygiene, but many resist such measures. Of these a goodly proportion will yield to percutaneous applications of the galvanic current, poles being applied to the hypogastric and lumbar regions, and a current of from 25 to 50 ma. being turned on without shock. But often we must resort to local treatment; and of the nature of the local treatment that is most appropriate I have had some very positive experience,—an experience, in fact, which led to the conception of the pathological condition advocated in this paper. Forcible dilatation certainly cures many cases, doubtless by paralyzing the irritable fibers, as in fissure of the anus, and by stimulating nutrition; but it is not a sovereign remedy in a large proportion of cases. In my experience, a more certain and less formidable remedy may be found in the intra-uterine action of one pole of a galvanic current,—usually the negative pole,—when a promotion of flow is desired, with a current varying from 15 to 50 ma. *pro re nata*. A few such applications, during one or two intermenstrual periods, has cured a number of cases in my hands. A typical case was that of a young French girl of twenty-four years, who had been menorrhalgic since puberty, becoming much worse during the year preceding her application for treatment. She was badly constipated, and I at first expected to relieve her by correcting this, but her next menstrual period was as bad as ever. Examination then showed a small uterus with healthy surroundings. The sound could not be passed beyond the internal os. Twenty-five milliampères, positive, were given for two minutes with the electrode in this position. Six days later the same instrument went to the fundus, without the use of a tenaculum, and forty milliampères were given. This was followed by an easier flow than for several years. Two similar applications were made during the next intermenstrual period, followed by a painless flow. Since then five menstrual periods have passed, all normal and free from pain.

Among my notes of married women treated in this way for menorrhalgia, three, who were apparently sterile, have become pregnant and borne children.

As contrasted with forcible dilatation this method is simple, does not require an anesthetic, and may be employed in young girls without the use of a speculum.

DISCUSSION.

Dr. A. Graydon.—I have at present under treatment a young girl who two years ago was operated upon by forcible dilatation. She is one of two typical cases, both of which were dilated. In the one case the dilatation was perfectly successful; in the other the operation did not effect the result expected. The girl still suffers severe pain at each period, and I have persuaded her to be put upon the electrical treatment. It is too soon to give the result. I have had curative results in sharp anteflexion, both with the faradic and with the galvanic current.

Dr. J. M. Baldy.—I have watched with a good deal of interest the electrical treatment as practised at the Pennsylvania and the Howard Hospitals. If we are going to get good results from electricity in any class of cases, we may probably expect to secure them in the class referred to by Dr. Massey.

The line is drawn sharply by Dr. Massey, between pathological anteflexion and normal anteflexion. In ninety-nine out of a hundred cases the anteflexion is perfectly normal. Many cases with an extreme degree of anteflexion, apparently pathological, have not the slightest symptom. In other cases without pathological anteflexion, or without any displacement, whatever, the dysmenorrhea is most severe. In many such cases I have obtained the best results by dilatation. So far as I have seen, electricity will undoubtedly relieve the pain in a certain number of cases. So will dilatation; but I take it that in the majority of cases dilatation will do the work better and quicker than electricity, and, perhaps, without as much annoyance to the patient.

I can illustrate this by reference to a case which was selected by Dr. Massey and myself as a test case. Dr. Massey thought that there was a pathological anteflexion; I thought that it was normal. She had suffered dysmenorrhea for years. She had come from a neighboring state, where she had had dilatation performed on a physician's table, without ether. We all know how unsatisfactory and incomplete that is. She had no premonitory symptoms at the menstrual period following the dilatation, and no pain during menstruation. The relief was absolute for three months. The pain then began to return, and she came to us one year afterward. She was given electrical treatment, 25 to 50 milliampères being used at two sittings. She suffered premonitory symptoms for ten days. The flow came on without pain, and continued two days without pain. She then had severe pain for one day, but during the rest of the period there was no trouble. She had two sittings, but the relief was only partial. With the dilatation she had one sitting, and this without ether, by no means as thorough as what we understand by rapid dilatation, and the relief was absolute for three months. If this woman were put under ether and full dilatation made, the relief, in all probability, would be absolute and permanent. The same result could not be accomplished without electricity under several months' treatment.

Dr. J. Price.—My experience scarcely corresponds with that of some of the gentlemen. I am not in the habit of dilating, nor have I passed a sound for some time, nor am I in the habit of jumping to a conclusion as to the necessity of treatment without recognition of a lesion. There is scarcely a married woman who has borne children, who has not suffered with dysmenorrhea, due to one of the conditions under consideration. If she had consulted a physician she would probably have been treated by dilatation or electricity, as few ever escape. This I could demonstrate by the history of the wives of young physicians. In many of these cases operation had been suggested, yet a few months later they married and became mothers. Without the presence of some positive pathological condition, I do not see why we should submit these women to operation, for surely the indications are not clear, and you have no trenchant argument for local treatment. The more I keep my hands off, the better am I satisfied with the results. From no small experience, I am satisfied that many of these women suffer serious troubles from too much treatment; some suffer from too much closure of cervix, too much dilatation, too much intra-uterine treatment, and too much electrical treatment, and later submit to abdominal section for no minor trouble in the pelvis, clearly due, in many cases, to vicious treatment. I can report a large number of sections done after closure of the cervix and after dilatation. I have removed large pus-tubes, one week, one month, one year, after dilatation, and after closure of the cervix.

I will present these specimens to fortify my remarks. This pus-tube, larger than the uterus, was treated for two years with a pessary and other forms of treatment. It was mistaken for a retroflexion. I

examine a large number of women, and fail to find pathological anteflexion, except in the presence of these conditions. If you find a pathological anteflexion, you will find a pathological lateral or bilateral trouble, which contra-indicates intra-uterine treatment. In the fourth volume of Pepper's "System of Medicine," a case is cited in which the cervix was closed, and the author of the article a few weeks later removed pus-tubes. A few days ago I removed a small dermoid tumor from a woman who had suffered for years. She lay in a hospital for eight months, and submitted to seventy applications of the actual cautery for sciatica, and yet no vaginal examination had been made. In this case there was a clear indication for examination.

Dr. William L. Taylor.—I fully agree with Dr. Price as to the number of cases of dysmenorrhea, but will also add the cases of young girls who are confined to bed every month for from one to four days. I would ask Dr. Price what is his treatment for such cases? These cases are numerous and require some rational treatment. We can divide cases of painful menstruation into two classes,—the one due to obstruction, the other to excessive ovarian congestion. In the first class dilatation, and in the second galvanism with counter-irritation, have given me good results.

Dr. J. Price.—I frankly say that I am afraid of the treatment, and have just reasons for being afraid. I wish to fortify the position of Dr. Goodell, one that he has held for a long time, although he practises these methods freely. Dr. Goodell has repeatedly called our attention to these moral rapes, and I wish to say that this is done too often, particularly by our female friends. Sex surely does not sanction the examination of every young girl with backache.

Dr. William L. Taylor.—That does not answer my question. There are a certain number of cases that are confined to bed every month, and in which the bromides, opium, and chloral are largely contra-indicated. The only method in such cases is to make an examination and use such positive operative measures as will relieve the trouble.

Dr. Joseph Hoffman.—I have under observation such a patient as Dr. Taylor has spoken of. I have treated her for a number of months, but have not yet made an examination. The girl is sixteen years of age, and has pain for three or four days at every period. I advise going to bed and the use of hot fomentations. This is done for two or three days, and for the rest of the month she does very well. Where the girl is anemic and neurotic, these conditions very likely lie at the bottom of the trouble. In these cases the best treatment is rest.

So far as intra-uterine applications are concerned, I think that there is no doubt that they are abused. Those who are called upon to treat painful micturition in males from bladder trouble, would not feel justified as soon as a man came with such disorder to pass a bougie at once. We are not to suppose because there is pain there is stricture. Pain may exist outside of any tangible condition, and may be cured by simple rest.

In regard to Dr. Massey's nomenclature, I see nothing in the terms suggested that has not been expressed before.

Dr. John C. DaCosta.—The gentlemen seem to consider dysmenorrhea a disease rather than a symptom. We have a variety of forms of dysmenorrhea. There may be an obstructive dysmenorrhea, a congestive dysmenorrhea, and a neuralgic dysmenorrhea. Take a sharply flexed uterus. If the canal follows a perfect curve, there is no dysmenorrhea. If, however, there is a sharp bend, such a uterus will have to go into a mimic labor to expel the blood, and we have dysmenorrhea. Again, the uterine canal may be large enough to pass the menstrual blood, but as the result of such passive or active congestion as may follow "catching cold" or the congestion sometimes following coition, there may be swelling of the mucous membrane which will close the canal, and the woman will suffer pain. There may be another form due to ovarian neuralgia. In the first two classes dilatation answers the purpose ; in the last, electricity may answer.

I cannot understand that peculiar formation of mind that is not afraid to cut open the abdomen, tear adhesions apart, and yet will not examine the pelvis by the vagina to see what is the matter, or is afraid to dilate a cervix. With all due deference to Dr. Price, I do *not* think that most cases of dysmenorrhea are due to pus-tubes. I have seen a number of cases in virgins who could not have had gonorrhea (which some allege as the cause of pus-tubes) and who did not have pus-tubes. I have not been afraid to handle these cases, but have treated and cured them, and there has been no after-trouble.

Dr. William Goodell.—As, in a certain sense, I have been the parent of dilatation in this city, I should like to give my experience. I have performed the operation three hundred and fifty-four times, and barring the first few cases in which I did not use antiseptic precautions, there has been no trouble, and even in one or two of the earlier cases where trouble occurred, it was very slight.

I agree with Dr. Price that young girls should not be examined without due reason ; but when a girl comes who has been suffering month after month, and year after year, and her life is made miserable, it is only right that something should be done to relieve her. I object strongly to the frequent vaginal examinations that are made simply for backache. Where an examination is desired by the mother, I frequently make a rectal examination; this enables me to determine the position of the womb and the condition of the pelvic organs.

I think that Dr. Price sees these things from a different point of view. He has a large dispensary practice, and sees a certain class of patients who are liable to gonorrheal trouble. He is probably right from his point of view. I have, myself, never seen a positive case of dysmenorrhea from pus-tubes. I have seen painful menstruation in these cases, but the pain was in the ovarian region. It is not a uterine pain. Ovaralgia is by no means frequent without pus-tubes.

I should like Dr. Price to explain one of his remarks. He speaks of cases of closed os uteri in which there were pus-tubes.

I cannot give up rapid dilatation of the cervical canals to electricity, unless the latter is as effectual, and it can be used without ether and at one sitting. The only objection to the operation of rapid dilatation is the necessity to give ether. A physician not long ago made the statement that he could cure any kind of stricture of the urethra by electrolysis. He was offered cases by some of his brother physicians of that city; but, if my memory serves me no trick, when he was closely challenged he failed to respond. I have seen the statement made in some of the medical journals, that the use of electricity is followed by dilatation of the uterine canal. But I do not understand the *rationale* of this agent, and I should like to hear Dr. Massey explain it.

Dr. J. Price.—I did not attribute dysmenorrhea to the presence of pus-tubes. What I said about pus-tubes had nothing to do with dysmenorrhea, or its treatment by dilatation or electricity, except as sequelæ. I wish clearly to condemn the indiscriminate examination of virgins. General treatment, out-door life, and rest does cure these cases. It is in just these cases that Dr. Weir Mitchell recommends his rest treatment, and in which he has such success. I would refer Dr. Taylor to the collection of photographs which were sent here by Dr. Playfair. I presume that all these women suffered dysmenorrhea, and they were all cured by rest, and, I venture to say, had no local treatment.

Dr. J. M. Baldy.—Does Dr. Price mean to say that all cases of dysmenorrhea in virgins can and should be cured by the rest treatment? I have heard him unqualifiedly condemn it.

Dr. William Goodell.—By what authority does Dr. Price state that the cases of Dr. Playfair suffered with dysmenorrhea.

Dr. J. Price.—I only presume that they did. They got well with general treatment, married, and bore children without electricity.

Such cases, in my experience, always have dysmenorrhea. But few women are free from it.

Dr. J. M. Baldy.—There is still the class of cases referred to by Dr. Taylor, of young girls who are not married, and have no idea of getting married, and who suffer the torments of the damned at the menstrual periods. They have to go to bed for three or four days each month, and some are made worse for going to bed. These cases surely demand some treatment. It is all very well to indiscriminately condemn electricity, dilatation, and other forms of local treatment, but what does Dr. Price offer us in their stead? Practically nothing. Dilatation will cure a certain number of these cases. Everyone admits that indiscriminate examinations are wrong; but the members of this society are surely beyond such an abuse. If a woman has been suffering for years, is still single, and getting worse, her virginity should be no bar to treatment.

Dr. William Goodell.—I have had a good many patients under the rest treatment, and while Dr. Price is right in regard to the majority of cases of dysmenorrhea cured by this treatment, yet he is not wholly right. I have been obliged to dilate some of my own cases, and I have repeatedly been asked by Dr. Mitchell to dilate patients for him who had been under the rest treatment, and who have been cured in every respect, with the exception of the dysmenorrhea.

Dr. M. Price.—There is one point I want to discuss, and that is the examination of virgins. A short time ago I sent a patient to a specialist for treatment of the throat. He told her he could cure her in six weeks; but at the end of that time she was no better. He then told her that she had uterine trouble, *he could see it in her throat*, and sent her to two or three specialists, who would not examine her, there being no indication for so doing. He said he would have her examined in spite of the father, and in spite of the opinions of the specialists. If it is ever necessary to examine a virgin it should be done under ether, in the presence of more than one individual.

Dr. G. Betton Massey.—Dr. Baldy has scarcely treated me with fairness in mentioning a case in which the treatment is only commenced; still his narrative shows considerable benefit from two applications. The paper did not claim that a single treatment would cure these cases. I distinctly stated "two or three treatments during at least two intermenstrual periods."

I am glad to see that, to-night, some of the surgeons are so conservative. While I agree with what has been said as to the inadvisability of vaginal examination in virgins, yet all must admit that at certain times examinations become necessary. I have, myself, always tried other measures first, and continued them for some time—often too long. A special advantage of this method of local treatment is that you do not have to use the speculum, and it is not necessary to dilate the vagina.

Dr. DaCosta has said that the menstrual pain is sometimes due to trouble in the ovary; I particularly excluded that from the paper. I believe it is pretty well understood that you can discriminate between the uterine pain of menorrhalgia and the ovarian pain preceding the sickness.

Dr. Goodell has made a good point in regard to the inefficiency of a single sitting of electricity. Where the trouble has to be relieved at a single sitting, dilatation comes in. I cannot explain how electricity overcomes the contraction of these canals. I have been unjustly charged with a mixing of facts in my book by one of the reviewers, who pointed out that in one place it is stated that there might be atresia of the cervix from the use of too powerful a current, and, in another place, the same thing is recommended to produce enlargement of the cavity. I have, myself, never seen atresia follow the use of heavy currents. Apostoli mentions its possibility, but he uses a sound that is bare at the external os, whereas, under the impression that a cauterization at the mouth is inadvisable, I try to apply the cauterization to a certain part only of the cavity. However it may be explained, I do not find that under the application of either the positive or negative current to the cavity of the uterus, after one or two applications the sound passes with greater ease, and that the succeeding menstrual flow is almost invariably less painful. There are two possible explanations: the one, that the canal is rendered patulous; the other, that spasm is relieved. I do not believe that pelvic trouble can be brought on by this treatment of menorrspasm in a woman otherwise healthy, and should not hesitate in such cases to apply powerful currents to the interior of the uterus, if proper aseptic measures were used. The troubles which may follow are due rather to dirt, than to the operation.

---

THE LAST case of murder under the name of "Christian Science Treatment" has aroused so much indignation in Massachusetts, that stringent legislation will be brought to bear to extirpate what is, without question, one of the most pernicious frauds and delusions that a community has ever been cursed with.—*Buffalo Commercial Advertiser.*

